# Supplementary material for: Correction of amblyopia in cats and mice after the critical period
Source: eLife. 2021 Aug 31;10:e70023. doi: 10.7554/eLife.70023 (PMC8456712; doi:10.7554/eLife.70023)
Supplement: Supplementary file 1. [file elife-70023-supp1.pdf]

Supplementary File 1: Table of monocular responses in mouse V1 after long-term MD (or sham) followed by fellow eye TTX (or saline).

(A) Deprived contralateral eye

			VEP magnitude (uV; **mean**± SEM)				
Group	*n*	Spatial freq. (cpd)	P47‡	P54	P61	P68	P75‡
Sham	16	0.05	**185.8** ± 17.20	**173.34** ± 18.92	**186.7** ± 20.08	**164.9** ± 15.91	**168.2** ± 15.95
		0.2†	**195.1** ±14.91	**182.6** ±13.12	**190.5** ±12.02	**191.9** ±15.70	**194.0** ±16.30
		0.4	**112.9** ± 13.54	**111.3** ± 12.31	**112.4** ± 11.94	**101.8** ± 10.39	**110.1** ± 9.690
MD	13	0.05	**113.1** ± 13.90	**129.6** ± 16.14	**128.8** ± 13.99	**96.57** ± 11.07	**109.1** ± 16.70
		0.2†	**105.2** ±21.89	**127.3** ±18.30	**132.7** ±19.31	**114.2** ±14.83	**116.1** ±18.48
		0.4	**41.45** ± 7.197	**66.54** ± 9.788	**74.77** ± 13.31	**64.08** ± 11.49	**61.81** ± 13.60
MD then TTX	13	0.05	**95.46** ± 13.05	**156.5** ± 20.40	**178.5** ± 21.87	**166.6** ± 21.76	**177.6** ± 25.15
		0.2†	**95.84** ±20.00	**187.4** ±25.83	**214.5** ±27.49	**218.1** ±23.39	**210.8** ±26.63
		0.4	**35.12** ± 7.723	**93.13** ± 17.76	**120.1** ± 19.95	**108.3** ± 17.68	**133.1** ± 26.37

†row represented in Figure 2B-C; ‡column represented in Figure 2D

(B) Fellow (non-deprived) ipsilateral eye

			VEP magnitude (uV; **mean**± SEM)				
Group	*n*	Spatial freq. (cpd)	P47‡	P54	P61	P68	P75‡
Sham	16	0.05	**90.68** ± 10.17	**89.55** ± 11.42	**112.1** ± 18.39	**83.90** ± 9.861	**76.86** ± 8.851
		0.2†	**91.36** ± 9.335	**84.99** ± 9.324	**105.6** ± 15.29	**89.56** ± 9.759	**74.67** ± 9.849
		0.4	**47.41** ± 7.675	**45.58** ± 6.386	**47.21** ± 8.373	**43.83** ± 4.630	**37.91** ± 5.341
MD	13	0.05	**111.7** ± 20.39	**75.77** ± 9.100	**83.25** ± 16.79	**62.41** ± 10.54	**57.46** ± 9.788
		0.2†	**118.717** ± 17.32	**73.60** ± 10.30	**85.13** ± 19.37	**61.93** ± 9.419	**64.09** ± 12.69
		0.4	**53.19** ± 9.23	**40.82** ± 7.91	**42.74** ± 9.553	**31.64** ± 4.460	**26.91** ± 4.213
MD then TTX	13	0.05	**113.1** ± 17.38	**123.4** ± 19.09	**94.74** ± 14.03	**79.69** ± 10.48	**84.23** ± 14.57
		0.2†	**130.2** ± 20.97	**103.7** ± 21.61	**102.7** ± 18.63	**82.75** ± 15.47	**83.52** ± 18.45
		0.4	**62.47** ± 14.99	**49.47** ± 11.63	**67.90** ± 14.17	**40.96** ± 7.122	**50.41** ± 11.37

†row represented in Figure 2E-F; ‡column represented in Figure 2G
