# Supplementary material for: Correction of amblyopia in cats and mice after the critical period
Source: eLife. 2021 Aug 31;10:e70023. doi: 10.7554/eLife.70023 (PMC8456712; doi:10.7554/eLife.70023)
Supplement: Supplementary file 2. [file elife-70023-supp2.pdf]

Supplementary File 2: Table ofmonocular responses in mouse V1 after long-term MD followed by fellow eye inactivation or reverse occlusion.

(A) Deprived contralateral eye

Group	*n*	Spatial freq. (cpd)	VEP magnitude (uV; **mean**± SEM)				
			P47‡	P54‡	P61	P68	P75‡
MD then TTX	14	0.05	**95.40** ± 14.80	**161.1** ± 21.79	**146.7** ± 17.42	**147.0** ± 16.20	**170.1** ± 16.06
		0.2†	**107.3** ± 18.19	**171.3** ± 20.68	**167.5** ± 19.29	**164.1** ± 14.18	**196.7** ± 16.32
		0.4	**42.59** ± 6.221	**92.04** ± 14.85	**107.1** ± 13.02	**98.51** ± 11.47	**109.6** ± 13.08
MD then RO	15	0.05	**86.71** ± 8.53	**154.0** ± 17.70	**116.8** ± 14.99	**95.24** ± 14.58	**88.95** ± 14.30
		0.2†	**104.4** ± 14.82	**166.8** ± 12.44	**128.5** ± 17.08	**127.3** ± 20.00	**97.30** ± 13.71
		0.4	**40.66** ± 6.209	**91.49** ± 11.45	**66.58** ± 11.02	**63.22** ± 12.89	**60.18** ± 9.32

†row represented in Figure 3B-C; ‡column represented in Figure 3D

(B) Fellow (non-deprived) ipsilateral eye

Group	*n*	Spatial freq. (cpd)	VEP magnitude (uV; **mean**± SEM)				
			P47‡	P54‡	P61	P68	P75‡
MD then TTX	14	0.05	**104.5** ± 9.991	**93.29** ± 12.95	**83.77** ± 13.28	**75.40** ± 11.95	**73.87** ± 9.574
		0.2†	**119.3** ± 13.18	**56.23** ± 5.962	**58.09** ± 12.73	**81.72** ± 19.80	**60.89** ± 11.35
		0.4	**66.24** ± 9.288	**30.58** ± 5.586	**27.40** ± 9.281	**42.14** ± 13.30	**35.18** ± 5.402
MD then RO	15	0.05	**96.99** ± 12.25	**85.50** ± 22.25	**71.12** ± 14.62	**59.28** ± 14.51	**41.92** ± 8.882
		0.2†	**96.90** ± 11.60	**52.57** ± 14.43	**51.58** ± 10.62	**38.81** ± 7.354	**34.27** ± 7.008
		0.4	**56.77** ± 7.647	**31.79** ± 4.858	**24.52** ± 4.910	**19.66** ± 4.196	**22.75** ± 4.001

†row represented in Figure 3E-F; ‡column represented in Figure 3G
