# Supplementary material for: Correction of amblyopia in cats and mice after the critical period
Source: eLife. 2021 Aug 31;10:e70023. doi: 10.7554/eLife.70023 (PMC8456712; doi:10.7554/eLife.70023)
Supplement: Supplementary file 3. [file elife-70023-supp3.pdf]

Supplementary File 3: Table of ocular dominance indices in cat V1 after long-term MD followed by fellow eye TTX across spatial frequencies.

(A) C474 (*Top,* right V1; *Bottom,* left V1)

		Ocular dominance index												
Spatial freq.	Temporal frequency	P30[†]	P51[‡]	P55[‡]	P65[‡]	P67[§]	P69[§]	P71[§]	P73[§]	P75[§]	P81[¶]	P83[¶]	P87[¶]	P102[¶]
0.05 cpd	2 Hz	0.064	-0.617	-0.497	-0.460	0.522	0.511	0.592	0.516	0.686	0.182	-0.061	-0.034	0.030
	control	-0.077	-0.123	-0.029	-0.044	-0.015	0.054	0.079	-0.063	0.073	0.022	-0.102	0.081	0.004
0.1 cpd	2 Hz[#]	0.052	-0.568	-0.607	-0.601	0.442	0.443	0.578	0.565	0.742	0.046	-0.142	-0.079	0.021
	control	-0.048	-0.046	-0.145	-0.026	-0.017	0.073	0.122	0.025	-0.018	-0.155	0.042	0.046	-0.008
0.5 cpd	2 Hz	-0.122	-0.513	-0.341	-0.380	0.245	0.035	0.286	0.179	0.500	0.040	-0.213	-0.099	0.040
	control	0.051	-0.035	-0.043	0.090	-0.078	-0.056	0.078	0.081	0.062	0.032	-0.071	0.035	0.028
grey	2 Hz	0.051	-0.093	-0.027	0.009	0.040	0.043	-0.030	-0.033	0.015	0.059	-0.041	-0.038	-0.103
	control	-0.131	-0.027	-0.013	-0.064	0.052	0.151	-0.028	0.025	0.083	-0.007	-0.094	0.064	0.008
0.05 cpd	2 Hz	0.106	-0.638	-0.510	-0.511	0.541	0.530	0.577	0.514	0.637	0.186	-0.061	-0.061	0.029
	control	-0.076	-0.130	-0.036	-0.034	-0.021	0.054	0.067	-0.080	0.071	0.021	-0.097	0.070	0.015
0.1 cpd	2 Hz[#]	0.074	-0.571	-0.616	-0.622	0.497	0.455	0.545	0.558	0.710	0.047	-0.151	-0.088	0.022
	control	-0.019	-0.036	-0.131	-0.038	-0.020	0.033	0.123	0.014	-0.069	-0.138	0.040	0.104	0.019
0.5 cpd	2 Hz	-0.151	-0.485	-0.366	-0.381	0.283	0.022	0.259	0.194	0.477	0.045	-0.206	-0.096	0.042
	control	0.026	-0.023	-0.044	0.061	-0.085	-0.039	0.058	0.091	0.053	0.048	-0.070	0.022	0.011
grey	2 Hz	0.039	-0.073	-0.028	-0.016	0.053	0.071	-0.045	-0.029	0.070	0.002	-0.051	-0.090	-0.102
	control	-0.123	0.017	-0.010	-0.040	0.031	0.127	-0.015	-0.005	0.040	0.005	-0.087	0.052	-0.013

[†]before MD; [‡]after MD; [§]during fellow eye inactivation; [¶]after fellow eye inactivation;
[#]row represented in Figure 4G and Figure 4—figure supplement 2

(B) C475 (*Top,* right V1; *Bottom,* left V1)

		Ocular dominance index													
Spatial freq.	Temporal frequency	P30[†]	P51[‡]	P53[§]	P55	P57	P59[§]	P61[§]	P63[§]	P65[§]	P67[¶]	P71[¶]	P79[¶]	P97[¶]	P101[¶]
0.05 cpd	2 Hz	-0.039	-0.461	0.418	0.023	-0.274	0.511	0.459	0.693	0.569	0.008	-0.022	0.000	-0.014	0.019
	control	-0.037	0.006	0.053	0.003	-0.038	-0.091	-0.062	-0.027	-0.067	0.075	0.070	0.042	0.146	0.051
0.1 cpd	2 Hz[#]	-0.055	-0.467	0.250	-0.081	-0.428	0.457	0.416	0.661	0.550	0.037	-0.031	0.069	0.007	-0.025
	control	-0.074	-0.045	-0.064	0.086	-0.104	0.080	0.067	0.165	0.117	-0.052	-0.014	0.036	0.033	0.008
0.5 cpd	2 Hz	-0.131	-0.393	-0.017	-0.154	-0.555	0.263	0.124	0.274	0.186	0.179	-0.158	0.205	-0.043	-0.062
	control	-0.006	-0.051	0.007	-0.002	0.050	-0.044	0.017	-0.044	0.034	0.055	-0.070	0.063	-0.061	0.072
grey	2 Hz	-0.074	-0.026	-0.038	0.044	0.058	0.001	0.020	0.055	0.107	-0.059	0.097	0.015	0.039	0.021
	control	-0.124	0.020	-0.008	-0.033	0.029	-0.047	-0.075	0.149	-0.055	-0.003	-0.031	0.062	-0.078	-0.061
0.05 cpd	2 Hz	-0.056	-0.469	0.383	-0.040	-0.297	0.515	0.455	0.699	0.569	-0.011	-0.020	0.003	-0.013	0.029
	control	-0.033	0.025	0.028	0.003	-0.052	-0.052	-0.024	-0.008	-0.067	0.068	0.072	0.035	0.076	0.021
0.1 cpd	2 Hz[#]	0.017	-0.479	0.268	-0.069	-0.433	0.450	0.414	0.665	0.550	0.013	-0.033	0.066	0.024	-0.019
	control	-0.023	-0.042	-0.087	0.084	-0.093	0.094	0.018	0.193	0.117	-0.073	0.003	0.034	0.057	-0.005
0.5 cpd	2 Hz	-0.122	-0.371	-0.048	-0.129	-0.553	0.293	0.139	0.279	0.186	0.158	-0.172	0.197	-0.016	-0.039
	control	-0.012	-0.045	0.000	-0.041	0.048	-0.035	0.034	-0.010	0.034	0.039	-0.038	0.050	-0.036	0.051
grey	2 Hz	-0.099	-0.016	-0.068	0.046	0.040	0.063	0.018	0.069	0.107	-0.007	0.065	0.013	0.035	0.025
	control	-0.143	0.037	-0.001	-0.022	0.073	-0.025	-0.084	0.120	-0.055	-0.033	-0.023	0.057	-0.051	-0.037

[†]before MD; [‡]after MD; [§]during fellow eye inactivation; [¶]after fellow eye inactivation;
[#]row represented in Figure 4G and Figure 4—figure supplement 2

(C) C476 (*Top,* right V1; *Bottom,* left V1)

Spatial freq.	Temporal frequency	Ocular dominance index P30[†]	P51[‡]	P59[‡]	P61[§]	P63[§]	P65[§]	P67[§]	P69[¶]	P79[¶]	P85[¶]	P101[¶]
0.05 cpd	2 Hz	0.034	-0.740	-0.571	0.633	0.677	0.520	0.724	0.114	-0.005	0.019	0.005
	control	-0.073	-0.065	0.033	0.044	0.038	-0.111	0.033	0.073	0.010	-0.152	0.041
0.1 cpd	2 Hz[#]	-0.058	-0.733	-0.610	0.525	0.617	0.519	0.646	0.066	-0.021	-0.038	-0.008
	control	0.009	-0.092	0.078	0.047	0.025	-0.022	-0.036	-0.009	0.205	-0.032	0.035
0.5 cpd	2 Hz	0.027	-0.700	-0.537	0.381	0.410	0.390	0.382	-0.001	0.144	-0.100	0.016
	control	0.039	-0.028	-0.053	0.031	-0.049	-0.098	-0.008	0.053	0.099	-0.023	-0.040
grey	2 Hz	-0.070	-0.002	-0.059	0.114	-0.078	-0.074	0.089	-0.019	0.175	-0.094	0.052
	control	-0.005	-0.147	-0.073	-0.012	-0.053	-0.046	0.145	-0.103	0.133	-0.087	0.032
0.05 cpd	2 Hz	-0.070	-0.754	-0.560	0.657	0.649	0.552	0.728	0.077	-0.049	0.026	0.010
	control	-0.037	-0.067	0.051	0.054	0.013	-0.091	0.018	0.075	0.036	-0.156	0.032
0.1 cpd	2 Hz[#]	-0.144	-0.744	-0.581	0.581	0.606	0.546	0.668	0.029	-0.096	-0.035	-0.007
	control	0.030	-0.093	0.069	0.061	0.014	-0.011	-0.020	0.004	0.222	-0.012	0.025
0.5 cpd	2 Hz	0.087	-0.699	-0.524	0.437	0.401	0.413	0.389	-0.011	0.113	-0.096	0.017
	control	0.059	-0.020	-0.057	0.077	-0.052	-0.087	-0.035	0.045	0.076	-0.021	-0.045
grey	2 Hz	-0.067	-0.022	-0.068	0.135	-0.098	-0.046	0.103	-0.025	0.183	-0.080	0.054
	control	0.003	-0.160	-0.067	0.011	-0.066	-0.021	0.107	-0.086	0.154	-0.085	0.018

[†]before MD; [‡]after MD; [§]during fellow eye inactivation; [¶]after fellow eye inactivation;
[#]row represented in Figure 4G and Figure 4—figure supplement 2

(D) C479 (*Top,* right V1; *Bottom,* left V1)

Spatial freq.	Temporal frequency	Ocular dominance index P30[†]	P51[‡]	P59	P61[§]	P64	P67	P69[§]	P71[§]	P73[§]	P75[§]	P82[¶]	P123[¶]
0.05 cpd	2 Hz	-0.004	-0.483	-0.286	0.683	-0.192	-0.166	0.609	0.593	0.700	0.640	-0.022	0.032
	control	0.033	0.019	0.037	-0.017	0.083	0.038	-0.050	-0.018	-0.111	0.059	0.082	0.049
0.1 cpd	2 Hz[#]	0.103	-0.547	-0.269	0.689	-0.143	-0.119	0.604	0.627	0.666	0.681	0.009	0.023
	control	0.005	-0.050	-0.007	-0.030	0.063	0.000	-0.078	0.072	-0.044	0.026	-0.063	0.054
0.5 cpd	2 Hz	-0.046	-0.451	-0.386	0.510	-0.362	-0.290	0.487	0.329	0.451	0.499	0.033	0.037
	control	0.047	0.001	-0.018	-0.027	0.070	-0.040	-0.069	-0.049	-0.021	-0.004	0.019	0.054
grey	2 Hz	-0.074	-0.043	0.024	-0.007	-0.008	-0.041	-0.048	-0.011	0.148	0.126	-0.121	0.098
	control	-0.129	0.108	-0.001	0.018	0.010	-0.012	-0.066	-0.062	0.003	0.069	-0.039	0.060
0.05 cpd	2 Hz	-0.058	-0.497	-0.278	0.647	-0.136	-0.123	0.588	0.550	0.652	0.643	-0.033	0.034
	control	-0.030	0.017	0.007	0.867	0.091	0.022	-0.085	-0.015	-0.081	0.066	0.072	0.045
0.1 cpd	2 Hz[#]	-0.029	-0.550	-0.260	0.649	-0.121	-0.088	0.571	0.598	0.640	0.674	0.017	0.023
	control	-0.014	-0.042	-0.015	0.872	0.094	0.003	-0.087	0.041	-0.024	0.054	-0.092	0.042
0.5 cpd	2 Hz	-0.079	-0.475	-0.378	0.492	-0.326	-0.225	0.442	0.303	0.409	0.483	0.068	0.033
	control	0.039	-0.007	-0.042	0.839	0.109	0.000	-0.104	-0.043	0.030	-0.032	0.056	0.061
grey	2 Hz	-0.012	-0.056	-0.017	-0.027	0.018	-0.056	-0.095	-0.012	0.096	0.153	-0.100	0.091
	control	-0.054	0.111	0.027	0.873	0.036	-0.016	-0.094	-0.057	-0.016	0.112	-0.063	0.048

[†]before MD; [‡]after MD; [§]during fellow eye inactivation; [¶]after fellow eye inactivation;
[#]row represented in Figure 4G and Figure 4—figure supplement 2
